# Satisfaction Levels of Medical Attendants at a Pakistani Emergency Department

**DOI:** 10.7759/cureus.7696

**Published:** 2020-04-16

**Authors:** Jibran Ashraf, Mujtaba Hassan, Qaiser Iqbal, Momina Naseer, Sikander Idrees, M Ali Khan

**Affiliations:** 1 Cardiology, National Institute of Cardiovascular Diseases, Karachi, PAK; 2 Critical Care, National Institute of Cardiovascular Diseases, Karachi, PAK; 3 Neurosurgery, Sindh Government Hospital, Karachi, PAK; 4 Gastroenterology, Jinnah Postgraduate Medical Centre, Karachi, PAK

**Keywords:** satisfaction, emergency department, attendants

## Abstract

Introduction

Healthcare services all over Pakistan are facing an ever-growing patient flow. Rapid urbanization and a population boom are mainly responsible for this phenomenon. This is most evident in the emergency department. Not only are the patients in dire need of medical management but they require it within a certain time frame lest it is too late. It is difficult in such situations to deliver satisfactory services.

Many studies have analyzed satisfaction levels in doctors, nurses, postgraduates, and patients in the emergency department. But little data is available on the satisfaction levels of attendants that accompany the patients most of the time. Attendants are an integral part of the doctor-patient relationship and their perspective may offer some insight into the shortcomings and issues afflicting the system, especially with regards to emergency medicine.

Aim

To evaluate the satisfaction levels of attendants of patients treated at the emergency department.

Materials and methods

This is a cross-sectional study, held from January 1 to June 31, 2018. Patient and attendant confidentiality were ensured. Written consent was taken in all cases. Attendants of patients treated at the emergency department that followed up at four weeks were given a simple questionnaire to fill. There were 10 questions in it, with a simple “Yes” or “No” answer.

A “Yes” answer carried one point while a “No” answer had zero points. Satisfaction levels were scored out of 10. Satisfaction levels were grouped as very satisfied (9-10 points), satisfied (7-8 points), partially satisfied/partially dissatisfied (5-6 points), dissatisfied (3-4 points), and very dissatisfied (0-2 points).

Results

A total of 688 patients followed up at four weeks, with their attendants willing to fill in the questionnaire. Mean satisfaction levels were 7.21 ± 4.59. Almost 60% of the attendants were either very satisfied or satisfied with their experience. Attendants were most satisfied with the cost, lab facilities, availability of medicines, and medical equipment. Time management was the most concerning factor for the attendants.

Conclusions

Attendants are mostly very satisfied or satisfied with their experience in the emergency department. About one-fifth are either very dissatisfied or dissatisfied.

## Introduction

Pakistan is experiencing a population explosion. The population is predicted to be 305 million by 2050; currently, it is just over 200 million [1]. The increased population has led to rapid urbanization, with a prediction of at least 50% living in major cities by the year 2030. As a result, this rapid change has put the healthcare system, which is predominantly a social system, under great stress. Resources are limited, unevenly distributed, and, unfortunately, not always available on time.

This scarcity of resources is more apparent in the emergency departments of all major hospitals in Karachi. With an ever-increasing patient load, teams are working longer and for more rigorous hours. The capacity of the doctors and staff to care for patients is compromised, and care providers struggle to meet international medical standards, even at Karachi’s finest institutes [2]. This also holds true for the rest of the country.

The cost of medicine and equipment, the availability of doctors, their ability to provide the highest level of care, the time given to each patient, and the availability of lab and radiological facilities are the major factors influencing the quality of care at emergency departments. In view of the increasing need for medical services and the lack of resources, new approaches and policies should be made. Research into this specific area is much needed to meet the challenge [3].

The researchers undertook to assess the overall medical care experience by studying the satisfaction levels of medical attendants at the busiest emergency department in Karachi. Satisfaction levels were assessed in a simple ‘Yes’ or ‘No’ manner. We chose to evaluate the attendants rather than the patients to spare the patients from undue stress in their time of medical need. This study was an entirely subjective analysis.

## Materials and methods

Attendants of the medical patients who presented at the emergency department were requested to fill in the questionnaire. They were given ample time and space and the choice to fill the questionnaire at the hospital or at home and bring it back at the next follow-up. Written consent was taken in all cases. Attendant and patient confidentiality were ensured.

Study design

Cross-sectional

Sampling technique

Consecutive, non-probability

Duration

Six months (January 2018 to June 2018)

Location

Outpatient department (OPD), Jinnah Postgraduate Medical Centre (JPMC), Karachi, Pakistan

Inclusion criteria

Male or female patients aged 14 years or older were eligible for induction into the study. Patients were initially treated at the emergency department for conditions requiring medical treatment only (e.g., diarrhea, pneumonia, hematemesis, cerebrovascular accident). There had to be a family member or guardian (attendant) willing to complete the follow-up questionnaire. Patients and attendants were required to follow up at four weeks.

Exclusion criteria

All surgical emergencies, including orthopedic, neurosurgical, urological, gynecological, trauma, and otorhinolaryngological emergencies, were excluded from the study. Medico-legal cases, such as poisoning, homicide, and assault cases, were not included. Terminally ill patients, patients undergoing chemotherapy or radiotherapy, all cardiac emergencies, burn victims, and patients under 14 years of age were also excluded from the study.

Primary outcome

The responses of the medical attendants rather than the patients were assessed in this study. The rationale behind this decision is that because of the grave nature of their emergencies, most patients may not be fully aware of their surroundings or even their own responses. In medical terms, they may not be aware of time, place, or person. However, the attendants are acutely aware of the situations and dangers of their patients. It would stand to reason that attendants are better judges of how the emergency team responds to an emergency.

Furthermore, medical attendants are required to carry out many duties, which include recording patients’ medical history, signing important documents, arranging or donating blood supplies, relaying messages to concerned family members, and receiving positive or negative news about the patients. Because anxiety and fear are natural responses from patients, attendants also endeavor to make difficult experiences reasonably satisfying for the patients. Being an attendant at an emergency department is no easy task.

A simple questionnaire with 10 straightforward questions was presented to the attendants at the four-week follow-up appointment after an emergency room visit at the outpatient department (OPD). All attendants were 18 years or older. All questions required a simple “yes” or “no” response. All positive answers carried a one-point value, and all negative answers carried zero points. A cumulative score out of 10 possible points was given.

Overall scores also corresponded to subjective satisfaction levels in the attendants. Therefore, satisfaction levels were further classified as five different subgroups. These were: very satisfied (9-10 points), satisfied (7-8 points), partially satisfied/partially dissatisfied (5-6 points), dissatisfied (3-4) points, and extremely dissatisfied (0-2 points. The questionnaire is shown in Table 1. Incomplete forms were excluded from the study.

**Table 1:** Questionnaire

Please select one response only: “Yes” or “No.”			
Q1.	Were you satisfied with the initial response time of the emergency team?	Yes	No
Q2.	Were you satisfied with the time taken by the senior doctor/registrar to attend to your patient?	Yes	No
Q3.	Were you satisfied with the evaluation and time given by the senior doctor?	Yes	No
Q4.	Were you satisfied with the availability of medicines?	Yes	No
Q5.	Were you satisfied with the availability of medical equipment needed for your patient (e.g., bed, station, catheters)?	Yes	No
Q6.	Were you satisfied with the lab facilities?	Yes	No
Q7.	Were you satisfied with the radiological facilities?	Yes	No
Q8	Were you satisfied with the behavior of the staff and doctors?	Yes	No
Q9.	Were you satisfied with the environment of the emergency department?	Yes	No
Q10.	Were you satisfied with the cost of the patient’s overall experience?	Yes	No

## Results

Six-hundred eighty-eight attendants who followed up with their patients completed the questionnaire in the six-month study period. The mean age of patients was 57.44 ± 17.46 years. The oldest patient was 91 years old, with a provisional diagnosis of ischemic stroke. The youngest patient was 15 years old, with a provisional diagnosis of diabetic ketoacidosis.

Provisional diagnosis of patients

Infectious diarrhea was the most common diagnosis in the study, followed by abdominal pain. Most patients had gastrointestinal or pulmonary diseases. These diagnoses do not represent the true nature and numbers of all the medical cases treated at the Jinnah Postgraduate Medical Centre (JPMC) emergency department but, rather, these represent the patients who chose to follow up at four weeks. A summary of the provisional diagnoses is shown in Table 2.

**Table 2:** Provisional diagnosis of patients following up at four weeks

Provisional Diagnosis	N (%)
Infectious diarrhea/food poisoning	117 (17.0%)
Abdominal pain	79 (11.48%)
Pneumonia	65 (9.44%)
Dengue/malaria	62 (9.01%)
Ischemic stroke	47 (6.83%)
Severe vomiting	40 (5.81%)
Hemorrhagic stroke	31 (4.50%)
Uncontrolled diabetes	28 (4.06%)
Non-infectious diarrhea	25 (3.63%)
Anemia	23 (3.34%)
Porto systemic encephalopathy	23 (3.34%)
Sepsis	20 (2.90%)
Hematemesis	19 (2.76%)
Pulmonary tuberculosis	17 (2.47%)
Chronic obstructive pulmonary disorder exacerbation	17 (2.47%)
Ascites/spontaneous bacterial peritonitis	17 (2.47%)
Acute pancreatitis	13 (1.88%)
Acute hepatitis	12 (1.74%)
Diabetic ketoacidosis	9 (1.30%)
Renal colic	9 (1.30%)
Acute hepatitis	5 (0.72%)
Hypoglycemia	5 (0.72%)
Addisonian crisis	4 (0.58%)
Urinary tract infection	4 (0.58%)
Acute kidney injury	2 (0.29%)
Tension pneumothorax	1 (0.14%)
Ulcerative colitis flare	1 (0.14%)
Anemic failure	1 (0.14%)
Drug reaction	1 (0.14%)
Total	688 (100%)

Primary outcome

The mean satisfaction level was 7.21 ± 4.59 corresponding to a “satisfied” outcome (see the Methods section). The highest percentage of satisfaction among attendants was for the cost of the experience. The lowest percentage was for the environment in the emergency department. Primary outcomes and satisfaction with respect to each question are shown in Table 3.

**Table 3:** Overall satisfaction levels and with respect to each question

Question	Attendants that answered “YES”
	N(%)
Were you satisfied with the initial response time of the emergency team?	490 (71.22%)
Were you satisfied with the time taken by the senior doctor/ registrar to attend to your patient?	415 (60.31%)
Were you satisfied with the evaluation and time given by the senior doctor?	429 (62.35%)
Were you satisfied with the availability of medicines?	572 (83.13%)
Were you satisfied with the availability of medical equipment needed for your patient (e.g. bed, station, catheters, etc.)?	633 (92.0%)
Were you satisfied with the lab facilities?	660 (95.93%)
Were you satisfied with the radiological facilities?	489 (71.07%)
Were you satisfied with the behavior of the staff and doctors?	524 (76.16%)
Were you satisfied with the environment of the emergency department?	347 (50.43%)
Were you satisfied with the cost of your overall experience?	631 (91.71%)
Overall satisfaction level (mean±standard deviation)	7.21 ±4.59

About 42% of the attendants were “satisfied” while approximately 18% were “very satisfied.” The diagnoses of hypoglycemia, hematemesis, diabetic ketoacidosis (DKA), dengue or malaria, and sepsis accompanied the best scores. The worst scores were recorded for ischemic and hemorrhagic strokes. Levels of satisfaction with respect to subgroups are shown in Table 4. Scores related to specific diagnoses are shown in Table 5.

**Table 4:** Satisfaction levels according to subgroups

Satisfaction level groups	N=688
Very satisfied	123 (17.87%)
Satisfied	289 (42.0%)
Partially satisfied/partially dissatisfied	145 (21.07%)
Dissatisfied	82 (11.91%)
Very dissatisfied	49 (7.12%)

**Table 5:** Satisfaction levels with respect to the provisional diagnosis

Provisional Diagnosis	Satisfaction levels (Mean ± Standard deviation)
Infectious diarrhea/food poisoning	7.81±2.33
Abdominal pain	6.21±3.50
Pneumonia	8.77±4.10
Dengue/malaria	9.22±0.89
Ischemic stroke	6.21±3.59
Severe vomiting	7.56±4.01
Hemorrhagic stroke	6.19±2.12
Uncontrolled diabetes	7.45±3.19
Noninfectious diarrhea	7.91±2.31
Anemia	8.65±1.59
Portosystemic encephalopathy	8.78±2.45
Sepsis	9.01±3.02
Hematemesis	9.31±2.93
Pulmonary tuberculosis	7.08±2.09
Chronic obstructive pulmonary disorder exacerbation	9.04±1.19
Ascites/spontaneous bacterial peritonitis	8.28±2.79
Acute pancreatitis	7.82±5.40
Acute hepatitis	7.53±1.04
Diabetic ketoacidosis	9.17±2.27
Renal colic	8.78±2.01
Acute hepatitis	7.65±4.12
Hypoglycemia	9.51±1.31
Addisonian crisis	7.32±3.48
Urinary tract infection	6.02±2.70
Others	7.85±2.98

## Discussion

The emergency department of JPMC treats over 1000 patients per day. Most health concerns involve the department of medicine, including gastroenterology, neurology, and nephrology. Keeping this in mind, the total of 688 patients who followed up during the six-month study seems low. This reality led the researchers to question why the follow-up rate was low, and they surmised several reasons.

Once treated for an emergency, patients prefer to follow up at their local medical center rather than the city’s central hospital. Many patients are referred to JPMC from other cities, and once they are discharged, they seek a follow-up appointment at their local centers. Because of the varied nature of emergencies, many patients follow up at OPDs specific to their diagnoses, which may be located at a different hospital or in a different department.

Three of the most common medical complaints at JPMC were diarrhea, with or without abdominal pain; severe pneumonia; and dengue. All of these resulted from infectious etiologies. All cases of diarrhea were linked to faulty sanitation or improper storage of food. Many cases occurred after patients ate at restaurants or street vendors. A few of these cases were eventually diagnosed as acute hepatitis and recorded separately.

While abdominal pain was associated with diarrhea in a large number of cases, other causes, such as acid peptic disease, gastrointestinal reflux disease, and food binging, were recorded as well. Abdominal pain was mostly treated conservatively with intravenous fluids and proton pump inhibitors. Pneumonia was seen mostly in elderly patients and was acquired in the community. The majority of cases responded to antibiotics.

Periodic outbreaks of dengue and malaria occur in the summer months in Pakistan, mainly due to the monsoons [4]. These incidents are very high for large metropolitan areas such as Karachi [5]. Dengue is a self-limiting disease that typically requires conservative management. Concomitant infections with malaria and symptoms such as a bone-breaking fever, hemodynamic instability, and bleeding require aggressive intervention [6-7]. Most patients presenting at the JPMC emergency department were considered high risk; however, the outcomes and mortality in this study were excellent.

Cerebrovascular accidents are frequently seen at emergency departments worldwide, and that reality proved true in this study. The majority of the remaining cases involved the complications of cirrhosis and tobacco smoking. Viral hepatitis is prevalent in Pakistan and was the leading cause of cirrhosis in our study. Tobacco smoking still plagues Pakistani society. Despite restrictions on the sale of cigarettes, smoking-related pulmonary diseases have remained steady.

Complications from diabetes are not uncommon [8]. The cases presented in this study do not represent the typical number and degree of diabetic cases at the JPMC emergency department. Rather, these were the patients who followed up at four weeks. In reality, the researchers evaluated other emergency cases who did not follow up. This is a common problem faced by physicians all over the country.

Overall satisfaction levels of medical attendants were reasonably good. Almost 60% of the attendants were either satisfied or very satisfied. Studies have been conducted in the last few decades analyzing the satisfaction levels of patients, nurses, pediatricians, doctors, and even postgraduates [9-10]. However, the data on attendants’ satisfaction levels are minuscule. One reason for this gap in the research is that doctors prefer self-initiated feedback over attendant-initiated feedback [11].

Attendants in this study were most satisfied with the cost, lab facilities, availability of medicines, and availability of medical equipment. One would expect such results in a social healthcare system in which these resources are available gratuitously. While radiological services are also provided by JPMC, they are limited by the availability of machines and trained staff for operating those machines. Thus, delays are often experienced in the radiology department.

Time is of the essence in the medical field. Doctors have to allocate enough time to each patient for optimal management while preparing to assess the next challenge. The first three questions on the questionnaire had to do with time, and reduced satisfaction levels for all three questions were highly indicative of the difficulty in time management and the stress the emergency teams are under constantly. These results were also highly suggestive of ever-increasing patient flow.

The lowest satisfaction levels were seen with regard to the environment of the emergency department. With over 1000 patients served each day, the environment can be overwhelming for attendants not accustomed to such a stimulating environment. Despite the reasonable separation between beds, it is inevitable that attendants hear or see other patients being treated. These distractions may lead to an uneasy feeling for the attendants despite good outcomes for their patients.

About 7.10% of the attendants were very dissatisfied. This study did not delve deeply into the factors that affected their experience. Perhaps, a more objective analysis is required to ascertain why these attendants had a dissatisfactory experience even though their patients survived the emergency room visits and participated in a four-week follow-up. No one factor could be singled out in our study in this particular aspect.

The highest levels of satisfaction were noted for the treatment of dengue or malaria, hematemesis, DKA, hypoglycemia, chronic obstructive pulmonary disorder exacerbation, and sepsis. Clearly, the severity of the medical emergency, as perceived by the attendants, affected the satisfaction level of the attendants. If the attendants perceived more serious health complaints by their patients, the attendants’ level of satisfaction was higher. Additionally, when patients responded to treatment in a shorter period of time, attendants scored higher on their level of satisfaction.

Concordantly, the lowest satisfaction scores were seen for the treatment of urinary tract infections and ischemic and hemorrhagic strokes. These conditions require conservative management and may take weeks or months to resolve completely. The outcome for strokes was rarely satisfactory for the attendants. Hemorrhagic strokes that warranted neurosurgical intervention were not included in the study.

Shortcomings

This study was limited by a number of shortcomings. "Lost to follow-up" was quite significant. The researchers did not take into account the number of times an attendant had visited the emergency department previously. Human bias could not be ruled out. Previous experiences by attendants at other medical centers were not accounted for. The duration of the illness (i.e., acute versus chronic) was not analyzed. By far, the biggest shortcoming was the lack of objective parameters.

## Conclusions

Most medical attendants were very satisfied or satisfied with their experience at the JPMC emergency department. The cost of care, lab facilities, availability of medicines, and availability of equipment contributed to the higher satisfaction levels. Time management, or its lack thereof, reduced the satisfaction levels. Further studies and more objective analyses are required to develop recommendations for changes to the emergency healthcare system as Pakistan’s population continues to increase.
